# Supplementary material for: From Semantics to Execution: Integrating Action Planning With Reinforcement Learning for Robotic Causal Problem-Solving
Source: Front Robot AI. 2019 Nov 26;6:123. doi: 10.3389/frobt.2019.00123 (PMC7805615; doi:10.3389/frobt.2019.00123)
Supplement: Supplementary file 1 [file Data_Sheet_1.pdf]

# Supplementary Material

## APPENDIX

### PDDL domain descriptions

For all planning domain definitions, we used only the STRIPS semantic requirement of the Planning Domain Definition Language (PDDL) (McDermott et al., 1998) , i.e., pre- and postconditions, and we realized the quantification operators by grounding the variables manually.

### A Block-stacking

The domain description of the block-stacking task is described in the following Listing 1.

**Listing 1.** Block-stacking domain

```
(define (domain block)
    (:objects o1 ... on)
    (:predicates
        (gripper_at ?o)
        (gripper_at_target)
        (at_target ?o)
        (on ?o1 ?o2)
    )
    (:action move_gripper_to_o
        :parameters (?o)
        :precondition ()
        :effect (and (gripper_at ?o) (forall ?o1 != ?o: (not (
           gripper_at ?o1))  (not (on ?o1 ?o)) (not (gripper_at_target
           ))))
    )
    (:action move_o_to_target
        :parameters (?o)
        :precondition (gripper_at ?o)
        :effect (at_target ?o)
    )
    (:action move_o_on_o
        :parameters (?o1 ?o2)
        :precondition (and (gripper_at ?o1) )
        :effect (and (on ?o1 ?o2)   (not (on ?o2 ?o1)))
    )
    (:action move_gripper_to_target
        :parameters ()
        :precondition ()
        :effect
        (and
            (gripper_at_target)
```

```
            (forall ?o: (not (gripper_at ?o))
            )
        )
    )
)
```

## B Tool use

The domain description of the tool use task is described in the following Listing 2.

**Listing 2.** Tool use

```
(define (domain tool)
    (:requirements :strips)
    (:objects obj rake)
    (:predicates
        (gripper_at ?o)
        (gripper_at_target)
        (at_target ?o)
        (at ?o1 ?o2)
    )
    (:action move_gripper_to_o
        :parameters (?o)
        :precondition ()
        :effect
            (and (gripper_at ?o)
                (forall ?o1 != ?o:
                    (not (gripper_at ?o1))
                    (not (at ?o0 ?o1))
                    (not (gripper_at_target))
                )
            )
    )
    (:action move_o_at_o
        :parameters (?o0 ?o1)
        :precondition (gripper_at ?o0)
        :effect (at ?o0 ?o1)
    )
    (:action move_o_to_target_by_o
        :parameters (?o1 ?o0)
        :precondition (and (at ?o0 ?o1) (gripper_at ?o0) )
        :effect (and (at_target ?o1) (at ?o0 ?o1) )
    )
    (:action move_o_to_target
        :parameters (?o)
        :precondition (gripper_at ?o)
        :effect (and (at_target ?o) (gripper_at ?o) )
```

```
    )
    (:action move_gripper_to_target
        :parameters (?o)
        :precondition ()
        :effect
        (and
            (gripper_at_target)
            (forall ?o:
                (not (gripper_at ?o))
            )
        )
    )
)
```

## C Ant navigation

The domain description of the ant navigation task is described in the following Listing 3.

For this listing we did not use the built-in PDDL objects and variables (indicated with ?<objname> syntax) to instantiate the predicates and actions. Instead, we implemented a script to generate the predicate and action definitions according to Listing 3 such that the following criteria are met:

1. Rooms (denoted <R>) are labeled 00, 01, 10, and 10, such that the 0 and 1 denote the column and row of the 2x2 grid in the ant navigation environment. E.g., room 00 is the lower left room and room 11 is the upper right room.
2. Doors (denoted <D>, the passages between the rooms) are labeled 0001 0010 0111 and 1011. The labels indicate the passages that connect the rooms. For example door 0001 connects room 00 with room 01.
3. For each door <D> and room <R> we generate the respective predicate names as listed in the `:predicates` section of the domain definition.
4. For each door and room combination we generate the action definitions indicated in the listing below, such that the connections of doors and rooms are appropriate. For example we generate an action definition `move_to_room_center_00_from_door_0001` because it is possible to move from door 0001 to the center of room 00. However, we do not generate the action `move_to_room_center_00_from_door_0111`, because door 0111 is not connected to room 00.

**Listing 3.** Ant navigation

```
(define (domain ant)
    (:requirements :strips)
    (:predicates
        at_door_<D>           ; whether the agent is at a door
        at_room_center_<R>    ; whether the agent is at a room-center
        at_target             ; whether the agent is at the target
        in_room_<R>           ; whether the agent is inside a room
        target_in_room_<R>    ; whether the target is inside the room
    )
```

```
    ; Move from room center of <R> to door <D>
    ; <D1> != <D> is the other door that is adjacent to <R>
    (:action move_to_room_center_<R>_from_door_<D>
        :precondition (at_door <D>)
        :effect (and
            (at_room_center <R>)
            (not (at_door <D1>)) )
    )

    ; Move to a door when inside a room that connects to the door
    (:action move_to_door_<D>_from_<R>
        :precondition (at_room_center_<R>)
        :effect (and (at_door_<D>) (not (at_room_center_<R>)) )
    )

    ; Move to the room center of <R> if not at a door <D1> or <D2> of
       that room
    (:action move_to_room_center_<R>
        :precondition (and (in_room_<R>)  (not (at_door_<D1>)) (not (
           at_door_<D2>)))
        :effect (at_room_center_<R>)
    )

    ; Move to the target within the room <R>
    (:action move_to_target_in_room_<R>
        :precondition (and (at_room_center_<R>)
                           (target_in_room_<R>))
        :effect (and (at_target) (in_room_<R>)
                     (not (at_room_center_<R>))
    )
)
```

## REFERENCES

McDermott, D., Ghallab, M., Howe, A., Knoblock, C., Ram, A., Veloso, M., et al. (1998). *PDDL - The Planning Domain Definition Language*. Tech. rep., Yale Center for Computational Vision and Control
